# A Late Miocene Accipitrid (Aves: Accipitriformes) from Nebraska and Its Implications for the Divergence of Old World Vultures

**DOI:** 10.1371/journal.pone.0048842

**Published:** 2012-11-09

**Authors:** Zihui Zhang, Alan Feduccia, Helen F. James

**Affiliations:** 1 College of Life Sciences, Capital Normal University, Beijing, China; 2 Department of Biology, University of North Carolina, Chapel Hill, North Carolina, United States of America; 3 Department of Vertebrate Zoology, MRC-116, National Museum of Natural History, Smithsonian Institution, Washington, D.C., United States of America; University of Lethbridge, Canada

## Abstract

**Background:**

Old World vultures are likely polyphyletic, representing two subfamilies, the Aegypiinae and Gypaetinae, and some genera of the latter may be of independent origin. Evidence concerning the origin, as well as the timing of the divergence of each subfamily and even genera of the Gypaetinae has been elusive.

**Methodology/Principal Findings:**

Compared with the Old World, the New World has an unexpectedly diverse and rich fossil component of Old World vultures. Here we describe a new accipitriform bird, *Anchigyps voorhiesi* gen. et sp. nov., from the Ash Hollow Formation (Upper Clarendonian, Late Miocene) of Nebraska. It represents a form close in morphology to the Old World vultures. Characteristics of its wing bones suggest it was less specialized for soaring than modern vultures. It was likely an opportunistic predator or scavenger having a grasping foot and a mandible morphologically similar to modern carrion-feeding birds.

**Conclusions/Significance:**

The new fossil reported here is intermediate in morphology between the bulk of accipitrids and the Old World gypaetine vultures, representing a basal lineage of Accipitridae trending towards the vulturine habit, and of its Late Miocene age suggests the divergence of true gypaetine vultures, may have occurred during or slightly before the Miocene.

## Introduction

Old World vultures are presently confined largely to Africa and Eurasia, and comprise one or two subfamilies. Three monotypic genera, *Gypaetus* (Lammergeyer or Bearded Vulture), *Neophron* (Egyptian Vulture), and *Gypohierax* (Palm-nut Vulture), are highly divergent from the remaining 6 genera and have been placed by some in a separate subfamily, the Gypaetinae [1]. The core 13 species, best known as the formidable large African and Asian vultures form the Aegypiinae. The fossil record of Old World vultures occurs in both the Old and the New Worlds as early as the Late Oligocene [2]–[11], and increases in richness up to the Pleistocene [12]. Among the great surprises of avian paleontology was that compared with the Old World, the New World has an unexpectedly diverse and rich fossil component of Old World vultures. Loye Miller described the first specimen as *Neophrontops americanus* in 1916, a form closely allied with the living Egyptian Vulture *Neophron percnopterus,* the smallest of the living Old World vultures, along with another Old World vulture *Neogyps errans*, both from the late Pleistocene Rancholabrean deposits [2]. Miller’s astonishment is clear in his later admission that “announcement was withheld for two years because of the wide geographic separation from other members of the Old World vulture group” [13]. Later, Howard, who studied the extensive and beautifully preserved material of Rancho la Brea, confirmed Miller’s appraisal, commenting that “the skeleton of *Neophrontops* is markedly like that of the recent Old World vulture, *Neophron*” [7], and that the differences between them “are of less note than those which exist between *Neopohron* and its contemporaries among the vultures today” [14]. As currently known, the fossil record of Old World vultures extends back to the Early Miocene of North America with *Palaeoborus* [3], and to the Late Oligocene of Europe with *Palaeohierax* [4], and is thus indecisive about the geographic origins of these birds. Rich summarized the poor fossil record of Old World vultures in the Old World, noting that in the past they had been reported in both the Old and the New Worlds [11], [15], but still no assessment of continental origins seemed advisable. Going back in time, the fossil record of the entire family Accipitridae in the Paleogene is extremely sparse [16]. Rich suggested that the North American accipitrid vultures might be polyphyletic [11], and new molecular evidence supports the hypothesis that the two extant clades of Old World vultures are of different evolutionary origin, with the Palm-nut Vulture *Gypohierax angolensis* as the earliest diverging lineage in Gypaetinae [17]–[20].

A tremendous early Late Miocene volcanic eruption in Idaho, some twelve million years ago, by some estimates as large as 100 times that of the Mount St. Helens eruption in Washington State in 1980, laid down an extensive ash fall over several hundred square kilometers, and a massive ash cloud traveled 1,600 km to the east in northern Nebraska [21], [22], where the ash was blown into certain low areas up to 2 to 3 meters thick. A remnant of the devastation included a remarkable death assemblage discovered near the town of Orchard, Nebraska, which included some 100 skeletons of the common short-limbed, large-bodied rhinoceros *Teleoceras major*, as well as dozens of horses, camels, and other large vertebrates, all comprising some 17 species. Of the mammal fossils, bone and teeth of *Teleoceras* are the most commonly found, and *anthoecia* from within the rhinoceros specimens indicate a diet of the grasses of the genus *Berriochloa*, a common late Neogene grass from the region [23]. The presence of siliceous grasses, along with evidence from symmetrically ripple-marked bedding planes of the ash and remains of aquatic turtles and diatoms, indicates the presence of some standing water typical of the known environment of *Teloceras*. This fauna is thus illustrative of the typical paleobiological setting of the Late Miocene of much of interior western North America [24], with a rich ungulate fauna, ecologically similar to that of modern east and central Africa, in a setting of warm, open savannas and wet grasslands. It was thus not surprising to discover that among the paleoavifauna were numerous specimens of a small species of the Crowned Crane *Balearica exigua* [25], which occurs today in wet grasslands of east and central Africa, and a long-legged accipitrid hawk *Apatosagittarius terrenus* [26], that converged on the living Secretarybird *Sagittarius*, a bird of sub-Saharan Africa that stalks its prey of snakes, other reptiles, and small mammals in open grassy savannas. The other bird recovered is an enigmatic accipitriform bird that appears to be somewhat morphologically intermediate between eagles and Old World vultures and is here described. The fossil material was originally studied in the late 1980s by AF, but publication was delayed until another independent assessment could be rendered. Following extensive re-study by ZZ and further evaluation by AF and HFJ, the new findings provide important clues to the origin and timing of the divergence of Old World vultures.

## Materials and Methods

### Nomenclatural Acts

The electronic edition of this article conforms to the requirements of the amended International Code of Zoological Nomenclature, and hence the new names contained herein are available under that Code from the electronic edition of this article. This published work and the nomenclatural acts it contains have been registered in ZooBank, the online registration system for the ICZN. The ZooBank LSIDs (Life Science Identifiers) can be resolved and the associated information viewed through any standard web browser by appending the LSID to the prefix “http://zoobank.org/”. The LSID for this publication is: urn:lsid:zoobank.org:pub:58CDF7BC-F588-4258-8C41-44569A5C7300. The electronic edition of this work was published in a journal with an ISSN, and has been archived and is available from the following digital repositories: PubMed Central, LOCKSS.

### Terminology and Material Examined

Osteological terminology follows that of Baumel and Witmer [27] and Howard [28]. The fossil materials are housed in the University of Nebraska State Museum (UNSM). All measurements were taken with calipers to the nearest 0.1 mm.

Comparisons with fossil material were made by actual fossils or adequate photos, with *Palaeohierax gervaisii*, *Palaeoborus howardae*, *Neophrontops americanus*, and *Neogyps errans*. Fossils were morphologically and biometrically compared with extant accipitriform skeletons (M, male; F, female) from the USNM (National Museum of Natural History, Smithsonian Institution): *Torgos tracheliotus* 347597M, 19990, 320977M, 321827F; *Trigonoceps occipitalis* 320859F, 347358; *Aegypius monachus* 614152, 428040F, 289569; *Gyps himalayensis* 19534M; *Gyps rueppellii* 430178; *Gyps coprotheres* 561314; *Gyps africanus* 587405, 431696, 430826M, 430014M, 431403F, 587404M, 431591F, 430016M; *Gyps fulvus* 227051F; *Neophron percnopterus* 17835; *Necrosyrtes monachus* 614886, 291442F, 18894, 620646, 291440M, 291443F, 291441F, 620645; *Gypaetus barbatus* 345684F, 17834, 343003; *Gypohierax angolensis* 224820, 291078F, 226143, 18892, 291316M; *Buteo jamaicensis* 290348M, 291203F, 610747F, 499645F, 641197F, 291202F, 634832F, 614355F, 553299F, 553822F, 637773M, 614358M, 614359M, 639916M, 614350M, 614353M, 614349M, 561859M, 614352F, 489995M, 500996M; *Buteo buteo* 614942M, 610367M, 603404M, 614944M, 289899M, 556290F, 556291F, 554270F, 610368F, 610369F, 605022F, 558450F, 614943F; *Buteo lagopus* 291309M, 322688M, 290440M, 291310F, 290438F, 290439F, 491026F, 491025F, 430284F, 499426F, 641200F, 641200, 18189, 322689, 613799; *Buteo regalis* 491843F, 320772M; *Buteogallus urubitinga* 345775M, 621696M, 319433M, 319432F, 345786F, 319431F, 343972, 343973, 345785; *Buteogallus aequinoctialis* 621851M, 621850M, 621849M, 344052F, 621054F, 621944F; *Buteogallus anthracinus* 562530M, 344053M, 613358M, 611555M, 612260M, 611554M, 612261F, 562529F; *Spizaetus cirrhatus* 344616M; *Busarellus nigricollis* 345773M; *Spilonis cheela* 562001; *Accipiter gentilis* 610353M; *Aquila rapax* 488148M; *Aquila audax* 344833, 226901, 620191, 430184, 620192M, 346461F; *Haliaeetus leucogaster* 560785F, 553664, 560784M, 556992M, 291360F; *Ichthyophaga humilis* 224807M; *Ichthyophaga ichthyaetus* 468555; *Circus cyaneus* 610732F, 610731F, 290353F, 289886F, 489908F, 499428F, 610734F, 290354M, 636773M, 291684M, 291173M, 291215, 347847, 18782, 17703, 319987; *Accipiter cooperii* 291170M, 290350M, 501627M, 636924M, 635130M, 291171M, 608265M, 621364F, 291177F, 614361F, 18197F, 553854F, 555720F, 498683F, 18041F, 612006F, 500998F; *Accipiter gentilis* 610347M, 290355M, 610349M, 290342M, 610351M, 610350M, 289977M, 322483M, 322686M, 610352M, 610354M, 291213M, 290339M, 322687F, 100400F, 499642F, 610359F, 610742F, 610358F, 622492F, 322684F; *Milvus migrans* 292039M, 319226M, 431663M, 291448M, 291788M, 291317M, 431662F, 319228F, 623327F, 557810F, 431664F, 346405F, 291789F, 319227F, 319489F, 318740, 603402, 18899, 17837.

## Results

### Systematic Paleontology

Aves Linnaeus, 1758 [29].

Accipitriformes Voous, 1973 [30].

Accipitridae Vieillot, 1816 [31].

Gypaetinae Vieillot, 1816 [31].

*Anchigyps voorhiesi* gen. et sp. nov.

urn:lsid:zoobank.org:act:39AA6AB3-9F4D-4C19-B548-9C15D4BB5E81 for *Anchigyps*, urn:lsid:zoobank.org:act:30113BCA-7C90-4FC8-A9D1-36FC71727528 for *Anchigyps voorhiesi*.

#### Holotype

**Figure 1:**
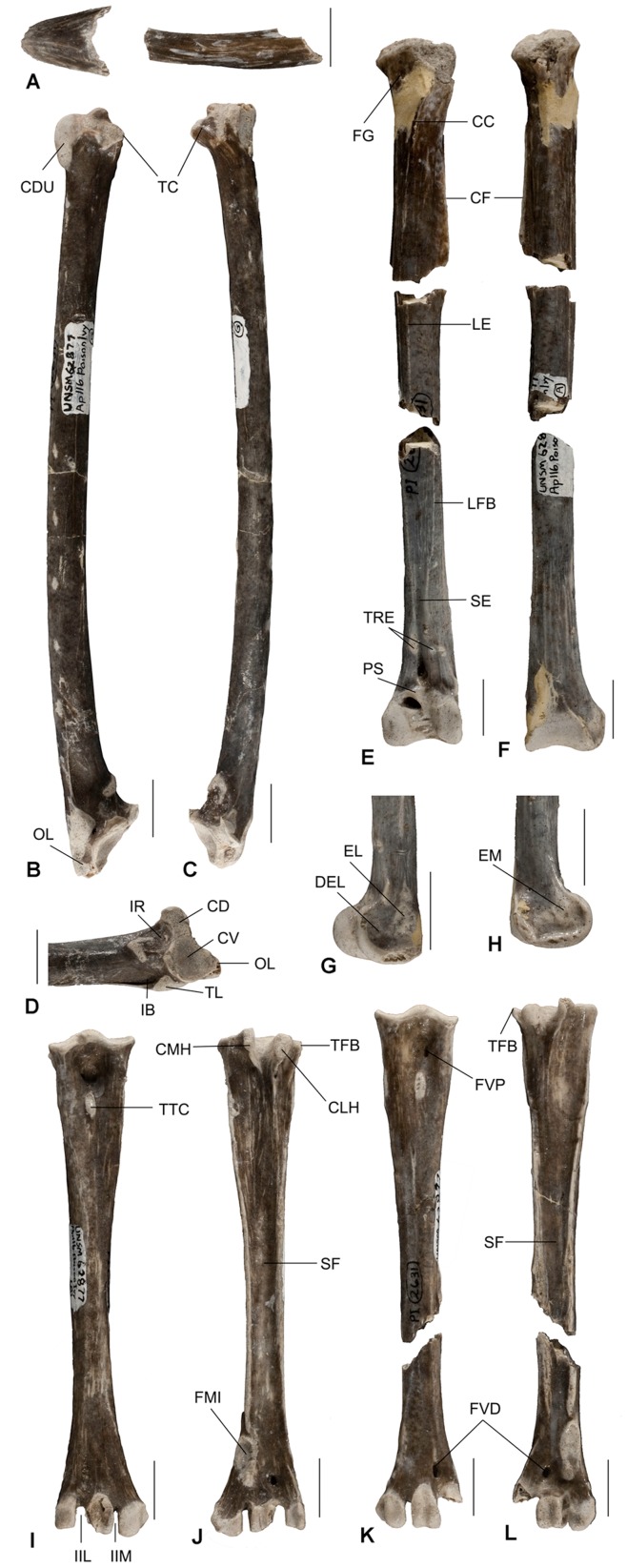
Holotype of *Anchigyps voorhiesi* gen. et sp. nov. (UNSM 62877). A, tip and part of the left ramus of the mandible. B–D, right ulna in ventral (B), dorsal (C) and cranial (D) views. E–H, left tibiotarsus in anterior (E), posterior (F), lateral (G) and medial (H) views. I–J, right tarsometatarsus in anterior (I) and posterior (J) views. K–L, left tarsometatarsus in anterior (K) and posterior (L) views. Abbreviations: CC, crista cnemialis cranialis; CD, cotyla dorsalis; CDU, condylus dorsalis ulnae; CF, crista fibularis; CLH, crista lateralis hypotarsi; CMH, crista medialis hypotarsi; CV, cotyla ventralis; DEL, depressio epicondylaris lateralis; EL, epicondylus lateralis; EM, epicondylaris medialis; FG, facies gastrocnemialis; FMI, fossa metatarsi I; FVD, foramen vasculare distale; FVP, foramina vascularia proximalia; IB, impressio m. brachialis; IIL,incisure intertrochlea lateralis; IIM, incisure intertrochlea medialis; IR, incisure radialis; LE, linea extensoria; LFB, linea m. fibularis brevis; OL, olecranon; PS, pons supratendineus; SF, sulcus flexorius; TC,tuberculum carpale; TFB, tuberculum m. fibularis brevis; TL, tuberculum lig. collateralis ventralis; TRE, tuberositas retinaculi extensori; TTC, tuberositas m. tibialis cranialis. Scale bars equal 1 cm.

Partial skeleton comprising a right tarsometatarsus missing part of the rim of the trochlea III, left tarsometatarsus lacking trochlea IV, right ulna, left tibiotarsus lacking part of the proximal end, and tip and part of left ramus of mandible (Fig. 1). Collection number UNSM (University of Nebraska State Museum) 62877.

#### Paratype

Left tarsometatarsus lacking the distal end and part of the hypotarsus. Collection number UNSM 62877.

#### Locality and horizon

Near the town of Orchard, Antelope County, Nebraska, in a 2 m thick bed of pure volcanic ash at the centre of NE quarter of NE quarter of NW quarter of Sec. 8, Township 28****N, range 7W**.** Cap Rock Member, Ash Hollow Formation, Late Clarendonian (early Late Miocene).

#### Etymology

*Anchi* from Greek meaning almost, plus *gyps*, vulture, in reference to its intermediate morphology between normal accipitrids and gypaetine Old World vultures. Species name is in honor of Michael R. Voorhies, who discovered the locality and led the excavations.

#### Diagnosis

Mandibular symphysis strong and broad; ramus relatively thin (narrow). Ulna relatively short, with absence of pneumatic foramina at both ends; condylus dorsalis joins shaft by abrupt transition. Tibiotarsus robust with a nearly circular transverse section at the central half; crista cnemialis cranialis long, directing outward proximally and distally reaching the proximal end of the crista fibularis; much lower epicondylus medialis anterior the front margin of the tibiotarsal shaft. Middle shaft of tarsometatarsus tapers abruptly; fossa infracotylaris dorsalis relatively narrow and deep; tuberculum m. fibularis brevis well developed; tuberositas m. tib. cranialis at median line of shaft; sulcus flexorius deep; all three trochleae terminate at same transverse plane; trochlea 3 slightly elevated and larger than the lateral and medial in distal view.

#### Measurements of holotype (mm)

Right tarsometatarsus: length, 84.5; width across trochleae, 15.8**;** greatest width at proximal end, 14.4**;** least width of shaft, 6.5. Left tarsometatarsus: length 82.6; greatest width at proximal end, 13.9; least width of shaft, 6.6. Right ulna: length, 127.5; greatest width at proximal end, 15.5; greatest width at distal end, 12.4; width (dorsoventral) of the middle shaft, 7.1; depth (craniocaudal) of the middle shaft, 5.9. Left tibiotarsus: preserved length, 118.3; greatest width at distal end, 13.3; greatest depth at distal end, 10.5; width of the middle shaft, 7.4; depth of the middle shaft, 6.8. Mandible: length of symphysis, 12.2; greatest width of symphysis 11.5; height of the proximal ramus, 5.6; width of proximal ramus, 0.9.

### Description

Mandiblar symphysis preserved with a length of 31 mm the proximal left ramus (Fig 1A). Symphysis length 12.2 mm, the width and the depth of the symphysis base is 11.5 mm and 5.5 mm, respectively. The symphysis is relatively robust and the shape resembling those of large- sized aegypiin vultures, but much different from the smaller *Gypohierax, Neophron* and *Necrosyrtes* which have relatively long and narrow symphyses, a reflection of a slender beak tip. Like extant Old World vultures, the mandibular ramus of *Anchigyps voorhiesi* is relatively deep and narrow, indicating an adaptation to greater loads in the dorsoventral direction and less resistance to lateral bending [32].

**Figure 2:**
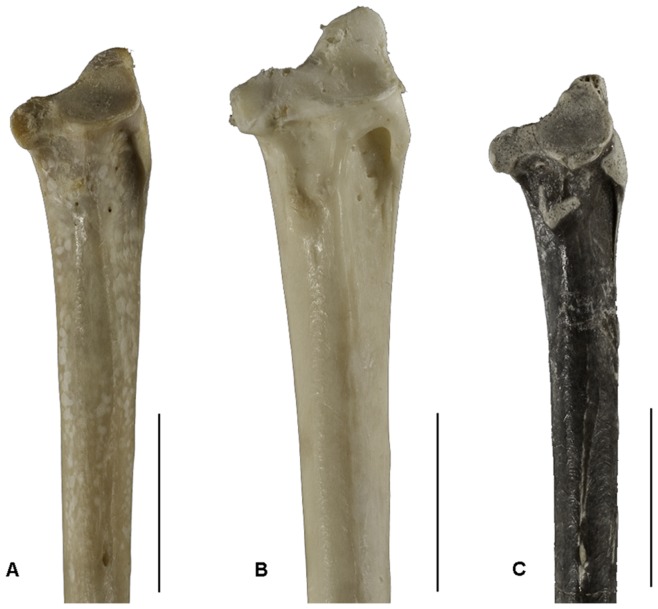
Comparison of the anterior view of the proximal end of the ulna. A, *Gypohierax*. B, *Necrosyrtes*. C, *Anchigyps voorhiesi* gen. et sp. nov., holotype (UNSM 62877). Scale bars equal 2 cm.

The right ulna (Fig. 1B–D) is completely preserved, and is relatively shorter (compared with relative lengths of tibiotarsus and tarsometatarsus) than in extant Old World vultures. The proximal end of the ulna is expanded dorsally by the processus cotylaris dorsalis, and proximally by the olecranon, which is strong, moderately long, and triangular in outline. There is no presence of a large excavation between the bases of the olecranon and the processus cotylaris dorsalis. Cotyla ventralis is larger and more concave than the cotyla dorsalis. Crista intercotylaris is low. Both the tuberculum lig. collateralis ventralis and the bicipital attachment are large and strong. The impressio m. scapulotricipitis is oval at the dorsal surface of the base of processus cotylaris dorsalis. Like *Gypohierax*, incisure radialis is slightly concave; the structure in extant Old World vultures is more excavated. The impressio m. brachialis is small, slightly concave and lacks pneumatic foramina as in gypaetine vultures (Figs. 1D, 2). The shaft of the ulna bends slightly caudally, with no definite longitudinal ridge between the facies caudodorsalis and facies caudoventralis. Papillae remigales ventrales is small and less protrudent than papillae remigales caudales. Dorsal to the papillae remigales caudales, there is a row of large, flat circular scars, probably for attachment of tectrices secoundariae dorsales majores. As indicated by the number of papillae remigales caudales and ventrales there are fewer secondary remiges in large eagles than in Old World vultures. Lineae intermusculares and a nutrient foramen are visible at the facies cranialis of the ulna. The distal end of the ulna is enlarged both craniocaudally and ventrodorsally to bear the tuberculum carpale, condylus dorsalis ulnae, and condylus ventralis ulnae. The tuberculum carpale is relatively large and more distinctly deflected from the shaft, as in all extant Old World vultures. Condylus dorsalis is large, elevated proximally, where it joins the shaft by an abrupt transition. A similar condition is seen in modern accipitrids apart from most Old World vultures whose dorsal condyle merges gradually with the shaft. Condylus ventralis is smaller than condylus dorsalis. Lying between the two condyles, the trochlea carpalis exhibits a shallow concave groove. The tuberculum carpale is large, more abruptly deflecting from the shaft, and there is an indistinct notch between it and the condylus ventralis. Depressio radialis is shallow. Incisure tendinosa is present, but shows no detail in morphology due to poor preservation. *Anchigyps voorhiesi* shares a feature with the gypaetine vultures and non-vulturine accipitrids in the absence of pneumatic foramina in the ulna; aegypine vultures show the presence of pneumatic foramina at varying parts of the proximal and distal end of the ulna.

**Figure 3:**
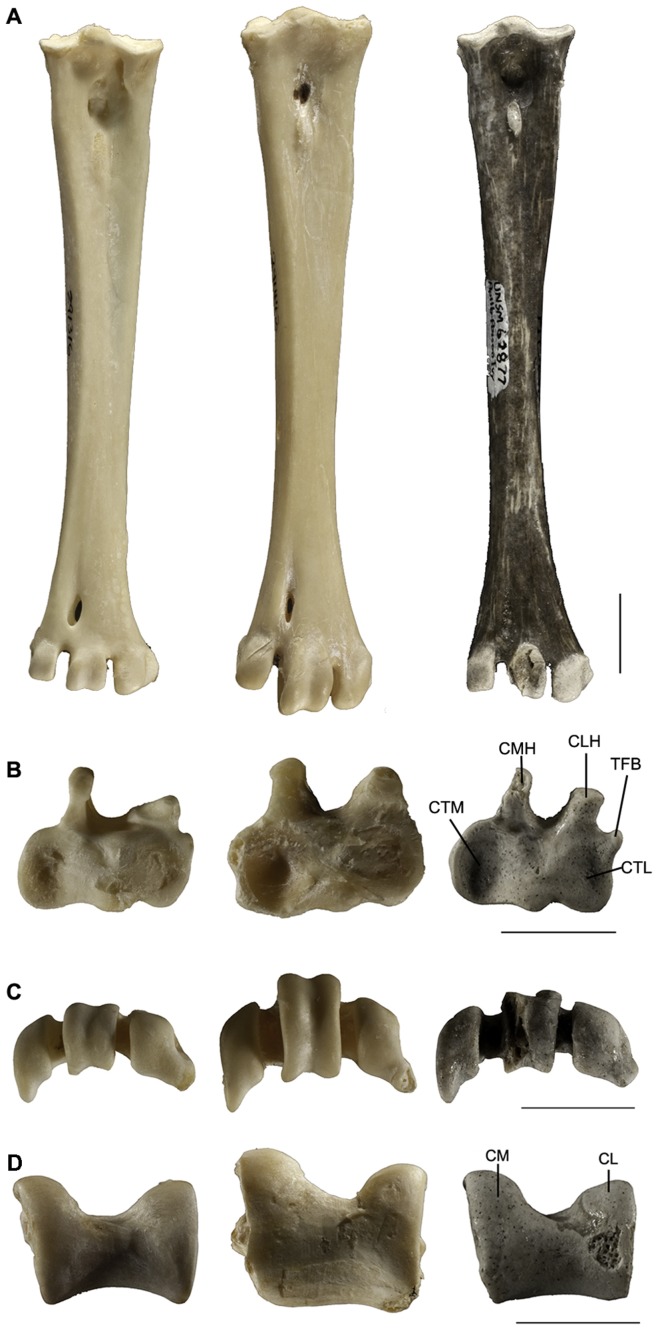
Comparisons of the tarsometatrsus and tibiotarsus of *Gypohierax* (left), *Necrosyrtes* (middle), and *Anchigyps voorhiesi* gen. et sp. nov., holotype (UNSM 62877) (right). A–C, right tarsometatrsus in anterior view (A), proximal view (B) and distal view (C). D, left tibiotarsus in distal view. Abbreviations: CL, condylus lateralis; CLH, crista lateralis hypotarsi; CM, condylus medialis; CMH, crista medialis hypotarsi; CTL, cotyla lateralis; CTM, cotyla medialis; TFB, tuberculum m. fibularis brevis. Scale bars equal 1 cm.

The left tibiotarsus (Fig. 1E–H) is nearly complete preserved except the proximal end. Crista cnemialis cranialis is relatively long, with its distal end reaching the proximal end of the crista fibularis (Fig. 1E), a feature of most non-vulturine accipitrids. Unlike modern large sized accipitrids, such as *Aquila*, the distal part of the crest is much lower and the proximal part directs outward in *Anchigyps voorhiesi*. The crest nearly parallels the shaft in extant Old World vultures, but only the distal part of the crest does so in *Anchigyps voorhiesi*. The crista cnemialis lateralis is as long as the cranial crest, although lower. Sulcus intercnemialis is narrow and shallow, judging from the direction and the morphology of the crests. Facies gastrocnemialis is relatively broad. Crista fibularis is long, bladelike, and more protrudent distally than proximally. The shaft of the tibiotarsus is straight, robust and nearly circular in cross section at the mid-point; this characteristic can be observed in most Old World vultures, but is absent in non-vulturine accipitrids. The distal end of the shaft is thicker anteroposteriorly than in non-vulturine accipitrids. Sulcus extensorius is narrow and deep, lies medial to the midline of the shaft, and is continuous with the canalis extensorius at the distal end. Pons supratendineus is short, weakly arched, and orients nearly perpendicular to the long axis of the shaft. The distal opening of canalis extensorius is located close to the base of condylus medialis. Proximal to the supratendinal bridge, two scars mark the attachment sites of the tuberositas retinaculi extensori; the medial one is much larger and located a little farther proximally than the lateral one. Linea extensoria is indicated on the crista cnemialis cranialis to the medial margin of sulcus extensorius. Linea m. fibularis brevis is relatively long, extending from the distal end of the crista fibularis to the lateral margin of the sulcus extensorius. The most distal end of the tibiotarsus expands significant medially. Tuberculum retinaculi m. fibularis is narrow and elongate at the lateral margin of the anterior surface of the shaft near the base of the condylus lateralis. Condylus medialis is identical in length with, and slightly wider both anteroposteriorly and mediolaterally than, the condylus lateralis. The outer surface of condylus lateralis (Fig.1G) is nearly round in outline. It has a shallow central excavation, the depressio epicondylaris lateralis, bounded by the heavy rounded margin of the condyle; this condyle projects farther anteriad than posteriad. The anterior surface of the condyle is flat, and slopes inward to merge with the perpendicular wall of the incisure intercondylaris. Epicondylus lateralis is large and low. Condylus medialis (Fig. 1H) of *Anchigyps voorhiesi* is similar to gypaetine vultures, protruding less anteriad than those of the aegypiins. The outer surface of the condylus medialis is relatively flat, without a distinct depressio epicondylaris medialis. Epicondylus medialis is low and lies slightly anterior to the cranial margin of the shaft, as in the small sized gypaetine vultures including *Gypohierax* and *Neophron*; it is well developed and situated in a line with the anterior margin of the shaft in the aegypiins and large eagles. In *Anchigyps voorhiesi*, the greatest width and depth at the distal end of the tibiotarsus are 13.3 mm and 10.5 mm respectively, and the ratio of these two dimensions (W/D) is intermediate to those of the aegypine and gypaetine vultures. The W/D ratio of aegypiins is smaller than that of gypaetines, thus indicating a much more sturdy distal tibiotarsus in the former group and a relatively flat tibiotarsus in the later group (Table 1, Fig. 3D). Although not preserved, the fibula is long, terminating 1 cm above the base of the condylus lateralis, with the spine strongly associated with the tibiotarsus as indicated by the presence of a long, clear ligamental prominence on the lateral surface of the distal tibiotarsus.

**Table 1:** Measurements of the distal end of the tibiotarsus.

	Greatest width (W) (mm)	Greatest depth (D) (mm)	W/D
*Anchigyps voorhiesi,* gen. et sp. nov.	13.3	10.5	1.27
*Gypaetus* (n = 3)	20.7 (19.0–21.6)	15.1 (12.8–16.3)	1.37
*Neophron*	14.4	10.7	1.35
*Gypohierax* (n = 5)	14.8 (14.3–15.3)	10.5 (10.1–11.0)	1.41
*Gyps himalayensis*	23.8	21.9	1.09
*G. rueppellii*	22.3	18	1.24
*G. coprotheres*	24.3	20.5	1.19
*G. fulvus*	25.4	22	1.15
*G.africanus* (n = 8)	21.4 (20.4–23.3)	18.5 (16.8–19.5)	1.16
*Torgos* (n = 4)	26.2 (25.2–27.6)	20.9 (19.9–22.2)	1.25
*Trigonoceps*	19.5	15.6	1.25
*Aegypius* (n = 3)	26.5 (25.0–27.6)	21.4 (20.4–22.5)	1.24
*Necrosyrtes* (n = 8)	14.5 (13.8–15.2)	11.7 (11.1–12.4)	1.24

Three tarsometatarsi are preserved: a left lacking the trochlea 4 (Fig. 1K–L), a right missing part of the rim of the trochlea 3 (Fig. 1I–J), and a left missing the distal end and part of the hypotarsus. The cotyla medialis is larger, more concave and slightly higher than the cotyla lateralis, a feature of all Old World vultures [33]. Eminentia intercotylaris is short and stout. Fossa infracotylaris dorsalis is relatively narrow, deep and contains foramina vascularia proximalia at the proximal end. The two equivalent foramina of *Anchigyps voorhiesi* are the most proximally situated among all Old World vultures examined. As in *Gypaetus*, the elongated tuberositas m. tibialis cranialis is present at the median line of the shaft in *Anchigyps voorhiesi*; this tubercle is slightly or obviously lateral to the midline in other Old World vultures and non-vulturine accipitrids, respectively. There is no impressiones retinaculi extensorii at the anterior surface of the proximal tarsometatarsus. In posterior view, the hypotarsus bears two protruding calcaneal ridges, which are separated by a deep sulcus hypotarsi. Crista medialis hypotarsi is slightly higher, longer and thinner than crista lateralis hypotarsi; the distal ends of both cristae tilt outward in proximal view. The lateral foramen vasculare proximale lies on the slope of the crista lateralis hypotarsi; it is on the lateral or the medial side in most of the Old World vultures and the bulk of accipitrids, respectively. Tuberculum m. fibularis brevis of *Anchigyps voorhiesi* is well developed, resembling a pillar, separated by a distinct notch from crista lateralis hypotarsi in proximal view (Fig. 3B), as in most non-vulturine accipitrids. In extant Old World vultures this structure is absent except in some specimen of *Gypohierax*, in which a very short, low ridge on the lateral margin of the posterior surface is present and might correspond to the tuberculum m. fibularis brevis in *Anchigyps voorhiesi* and most non-vulturine accipitrids (Fig. 3B). In comparison with extant aegypine vultures, the shaft of the tarsometatarsus of *Anchigyps voorhiesi* is relatively slender, flaring at both ends and tapering abruptly in the middle half (Figs. 1I–L, 3A). The shaft of the middle part of the bone is nearly triangular in cross-section, with the anterior, irregular surface sloping toward the medial margin. Sulcus extensorius lies on the anterior surface of the proximal half of the bone, and more clearly indicated than in extant Old World vultures. The lateral surface is flat, slightly concave at the center and rounded toward anterior and posterior margins. The posterior surface of the shaft in *Anchigyps voorhiesi* bears a distinct and well-developed sulcus flexorius as in *Gypohierax* and most non-vulturine accipitrids. The distal end of the shaft is nearly flat, with a less distinct fossa supratrochlearis plantaris. The foramen vasculare distale is oval, continuous with a short and shallow distal extensor groove at the proximal margin.

The distal ends of three trochleae lie in nearly in the same transverse plane, a feature similar to that of *Gypohierax*, but quite different from those of other Old World vultures where trochlea IV is much smaller and farther proximally situated than trochleae II and III (Fig. 3A). The wing of the trochlea for digit II, serving to hold in place the tendons of the muscles flexing digit II [34], is small as in most Old World vultures; the structure is usually long and protrudes more medially rather than posteriorly in most non-vulturine accipitrids. Incisure intertrochlea medialis of *Anchigyps voorhiesi* is narrower than incisure intertrochlea lateralis, as in the *Gypohierax*. Trochlea III is slightly larger than the medial and lateral trochleae. The anterior surface of the middle trochlea is a little more elevated than those of the other two, and merges gradually with the shaft of the tarsometatarsus in the proximal end. In general, the size and shape of trochlea III in *Anchigyps voorhiesi* is most similar to that of *Gypohierax*; whereas other Aegypiinae are characterized by having a much larger and more highly raised trochlea III (Fig. 3C), thus indicating a much stronger middle toe. The anterior middle groove of trochlea III is in line with the longitudinal axis of the tarsometatarsus as in all Old World vultures. Trochlea IV is slightly smaller and narrower than trochleae II and III; the outer edge gives rise to a short, posteriorly projecting wing, about equal to that on trochlea II, by which to hold the flexor tendons in place. Fossa metatarsi I is elongated and lies on the posterior surface, whereas it is in the inner posterior corner of the tarsus in most accipitrids; its much lower position suggests a relatively short metatarsal I in *Anchigyps voorhiesi*.

## Discussion

*Anchigyps voorhiesi* sp. nov. shares numerous osteological features with the Old World vultures, as follows: a very narrow ramus of the mandible, a nearly circular cross-section of the tibiotarsus shaft, a relatively large tuberculum carpale at the distal end of the ulna, a larger and slightly higher cotyla medialis compared with cotyla lateralis, a long fibula, and a relatively thick and convex lateral half of the proximal posterior surface of the tarsometatarsus, a short medioposteriorly projecting wing of trochlea II, as well as a short posterior wing of trochlea IV. Old World vultures are represented by two subfamilies, the Gypaetinae and Aegypiinae. *Anchigyps voorhiesi* is assigned to Gypaetinae rather than to Aegypiinae in important features, including the relatively smaller and less concave impressio m. brachialis, the absence of pneumatic foramina at the both ends of the ulna, a less anterior convexity of condylus medialis and a much more anterior position of the epicondylus medialis of the tibiotarsus. Among Gypaetinae, the new bird much resembles *Gypohierax* in the strong development of tuberculum m. fibularis brevis and sulcus flexorius, the similar size and morphology of the front trochleae and the relatively much lower position of fossa metatarsi I. Compared with the new bird, *Gypohierax* has a much more slender symphysis of the mandible, a short crista cnemialis cranialis, a relatively much longer ulna and much wider tarsometatarsus. These differences indicate that, although apparently not far removed phylogenetically, *Anchigyps voorhiesi* cannot be confidently placed in the genus *Gypohierax*. *Anchigyps voorhiesi* resembles the non-vulturine accipitrids in the presence of a well-developed tuberculum m. fibularis brevis and sulcus flexorius, a long although low crista cnemialis cranialis, absence of pneumatic foramina at the proximal and distal ends of the ulna, and the nearly coequal length and height of the three front trochleae. These features suggest *Anchigyps voorhiesi* belongs in the same clade as Gypaetinae but lacks some of the “vulturine” osteological characters that define the extant taxa.

In contrast to *Palaeohierax gervaisii,* which was found from the late Oligocene of France, and has been described as most similar to the living Palm–nut Vulture *Gypohierax angolensis*, the tarsometatarsus of *Anchigyps voorhiesi* is quite distinct in many aspects, characterized by having a relatively slender shaft, the much lower position of fossa metatarsi I, the absence of impressiones retinaculi extensorii, much highly raised calcaneal ridges, less developed projecting wing of trochleae II and IV. It is also clear that the lateral foramen vasculare proximale of *Anchigyps voorhiesi* lies on the slope of the crista lateralis hypotarsi, but it is on the medial side in *Palaeohierax.* The tarsometatarsus of *Anchigyps voorhiesi* agrees with that of *Palaeoborus howardae* in general conformation of trochleae, but fossa metatarsi I located slightly more distad, shaft tapers distally more abruptly, sulcus extensorius less excavated, sulcus flexorius more well-developed. The impressio m. brachialis of ulna is more concave in *P. howardae*. The main differences between *Anchigyps voorhiesi* and another small vulture, *Neophrontops americanus*, lie in the size of trochlea III and the proportion of limb elements. Trochlea for digit 3 is more robust than those for digits 2 or 4 in *N. americanus*, but slightly larger than the medial and lateral trochleae in *A. voorhiesi.* The ulna is obviously shorter in the new fossil. Comparisons of the distal end of the tarsometatarsus with *Neogyps errans* show neither the size of the wing of trochlea II, nor the height and breadth of trochlea III relative to those for digits 2 or 4, are obviously larger than in *Anchigyps voorhiesi.* The proximal tarsometatarsus of *Neogyps* is different from *A. voorhiesi* in the absence of tuberculum m. fibularis brevis, and the more distal and slightly lateral location of tuberositas m. tibialis cranialis.

The length of the ulna, tibiotarsus and tarsometatarsus of *Anchigyps voorhiesi* is nearly identical to that of the common North American Red-tailed Hawk *Buteo jamaicensis.* The relatively short ulna suggests that, compared with extant Old World vultures, the new bird was less proficient at soaring, and more proficient at flapping flight, by analogy with small to medium sized non-vulturine accipitrids. Additional support is the absence of pneumatic foramina of the ulna. Higher pneumaticity in the aegypiins as well as New World vultures is an adaptation for advanced soaring flight, whereas flapping accipitrines exhibit the least degree of pneumaticity [34], [35]. In *Anchigyps voorhiesi*, the well-developed tuberculum m. fibularis brevis, and a notch between it and the crista lateralis hypotarsi indicate a better development of the flexor tendon of the hallux, and thus a stronger control of the hind toe as in non-vulturine accipitrids. Its ability to flex the toes appears to be great, as in predatory accipitrids, by the presence of a distinct and deep sulcus flexorius, a structure in accommodating the bundle of tendons of the flexor muscles of the digits [34]. The strong hind toe and the powerful flexion of the toes are suitable for catching or killing prey, suggesting that *Anchigyps voorhiesi* may have been in part a predatory bird, but the strong symphysis and the relatively narrow ramus of the mandible reveal it to be a carrion feeder [32], perhaps using its grasping feet for handling food items. This would not be surprising given the rich species diversity of the megafauna in the Late Miocene of North America that provided an abundant food resource. The mosaic pattern of morphology relating to dietary preference apparently reflects that *Anchigyps voorhiesi* was not an obligate predator or scavenger. Predatory species at the Late Pleistocene Rancho la Brea also scavenged frequently [32].

Morphological, karyological and molecular studies strongly support the hypothesis that Old World vultures are polyphyletic, from two different evolutionary lineages [33], [35]–[38], although little can be ascertained about their place of origin. Compared with the more generalized scavengers of the Aegypiinae, the vultures of the Gypaetinae are characterized by a stronger grasping ability of the foot, and relatively more divergence in dietary diversity. The Gypaetinae is typically positioned near the base of the Accipitridae cluster, indicating an evolutionarily old lineage [17], [19], [38]. The unique Palm-nut Vulture *Gypohierax angolensis* of Africa has been thought to represent a transitional form from eagles to vultures [39]. *Anchigyps voorhiesi* reported here might represent a basal member of Gypaetinae in possessing a grasping foot and carrion-feeding adaptations, which have been regarded as characteristic of basal Accipitridae [35]. This finding provides additional important clues to the timing of the divergence of gypaetine vultures, possibly during the Oligocene to Miocene. The strong representation of Old World vultures among New World fossils is still another cautionary note on avian biographical conclusions based on extant avifauna; birds fly, and their ancestral distributions may not correlate with their modern distributions. The multiple lineages of diverse “vultures,” including the New World cathartids and two lineages of Old World vultures, suggest the ease by which natural selection may act to produce such iterative lineages of scavengers with vulturine habits and morphology in response to ecological conditions that produce abundant carrion, as in the North American Neogene. Given the occurrence of massive homoplasy and convergence in avian lineages future whole genome comparisons may be able to ascertain whether the currently classified gypaetine vultures actually represent a true clade or multiple lineages of vulturine accipitrids.

## References

[1] Mundy P, Butchart D, Ledger J, Piper S (1992) The vultures of Africa. London: Academic Press. 460 p.

[2] Miller LH (1916) Two vulturid raptors from the Pleistocene of Rancho la Brea. University of California Publications in Geology 9: 105–109.

[3] Miller AH, Compton LV (1939) Two fossil birds from the lower Miocene of South Dakota. Condor 41: 153–156.

[4] Brodkorb P (1964) Catalogue of fossil birds: part 2 (Anseriformes through Galliformes). Bulletin of the Florida State Museum, Biological Sciences 8: 195–335.

[5] Wetmore A (1936) Two new species of hawks from the Miocene of Nebraska. Proceedings United States National Museum 84: 73–78.

[6] Wetmore A (1943) Two more fossil hawks from the Miocene of Nebraska. Condor 45: 229–231.

[7] Howard H (1966) Two fossil birds from the Lower Miocene of South Dakota. Los Angeles County Museum of Natural History Contributions to Science 107: 1–8.

[8] Emslie SD (1985) The late Pleistocene (Rancholabrean) avifauna of Little Box Elder Cave, Wyoming. Contributions to Geology, University of Wyoming 23: 63–82.

[9] Hou L (1984) The Aragonian vertebrate fauna of Xiacaowan, Jiangsu-2. Aegypinae (Falconiformes, Aves). Vertebrata PalAsiatica 22: 14–20. [In Chinese].

[10] Zhang Z, Zheng X, Zheng G, Hou L (2010) A new Old World vulture (Falconiformes: Accipitridae) from the Miocene of Gansu Province, northwest China. Journal of Ornithology 151: 401–408.

[11] Rich PV (1980) “New World vultures” with Old World affinities? : A review of fossil and recent gypaetinae of both the Old and the New World. Contributions to Vertebrate Evolution 5: 1–115.

[12] Feduccia A (1999) The origin and evolution of birds, 2nd Edition. New Haven and London: Yale University Press. 466 p.

[13] Miller LH, DeMay IS (1942) The fossil birds of California: an avifauna and bibliography with annotations. University of California Publications in Zoology 47: 47–142.

[14] Howard H (1932) Eagles and eagle-like vultures of the Pleistocene of Rancho La Brea. Washington: Carnegie Institution of Washington Publication 429. 82 p.

[15] Rich PV (1983) The fossil history of vultures: a world perspective. In: Wilbur SR, Jackson JA, editors. Vulture biology and management. Berkeley: University of California Press. 3–25.

[16] Mayr G (2009) Paleogene fossil birds. Berlin: Springer-Verlag. 262 p.

[17] Seibold I, Helbig AJ (1995) Evolutionary history of New and Old World vultures inferred from nucleotide sequence of the mitochondrial cytochrome b gene. Philosophical Transactions of the Royal Society of London B 350: 163–178. 10.1098/rstb.1995.0150 8577858

[18] Wink M, Sauer-Gürth H (2004) Phylogenetic relationships in diurnal raptors based on nucleotide sequences of mitochondrial and nuclear marker genes. In: Chancellor R, Meyburg BU, editors. Raptors worldwide. Berlin: World working group on birds of prey and owls. 483–498.

[19] Lerner HR, Mindell DP (2005) Phylogeny of eagles, Old World vultures, and other Accipitridae based on nuclear and mitochondrial DNA. Molecular Phylogenetics and Evolution 37: 327–346. 15925523 10.1016/j.ympev.2005.04.010

[20] Griffiths CS, Barrowclough GF, Groth JG, Mertz LA (2007) Phylogeny, diversity, and classification of the Accipitridae based on DNA sequences of the RAG-1 exon. Journal of Avian Biology 38: 587–602.

[21] Voorhies MR (1981) Ancient ashfall creates a Pompei of prehistoric animals. National Geographic Magazine 159: 66–75.

[22] Voorhies MR (1985) A Miocene rhinoceros herd buried in volcanic ash. National Geographic Society Research Reports 19: 671–688.

[23] Voorhies MR, Thomasson JR (1979) Fossil grass anthoecia within Miocene rhinoceros skeletons: diet in an extinct species. Science 206: 331–333. 17733681 10.1126/science.206.4416.331

[24] Webb SD (1977) A history of savanna vertebrates in the New World. I: North America. Annual Review of Ecology and Systematics 8: 355–380.

[25] Feduccia A, Voorhies MR (1992) Crowned cranes (Gruidae: *Balearica*) in the Miocene of Nebraska. Natural History Museum of Los Angeles County Science Series 36: 239–248.

[26] Feduccia A, Voorhies MR (1989) Miocene hawk converges on secretarybird. Ibis 131: 349–354.

[27] Baumel JJ, Witmer LM (1993) Osteologia. In: Publications of the Nuttall Ornithological Club Baumel JJ, King AS, Breazile JE, Evans HE, Van den Berge JC, editors. Handbook of avian anatomy: nomina anatomica avium. 23: 45–132.

[28] Howard H (1929) The avifauna of Emeryville shellmound. University of California Publications in Zoology 32: 301–394.

[29] Linnaeus C (1758) Systema Naturae. 10th edition. Laurentii Salvii: Holmiae. 824 p.

[30] Voous K (1973) List of recent Holarctic bird species non-passerines. Ibis 115: 612–638.

[31] Viellot LJP (1816) Analyse d’une nouvelle ornithology élémentaire. Paris: Déterville. 70 p.

[32] Hertel F (1995) Ecomorphological indicators of feeding behavior in recent and fossil raptors. Auk 112: 890–903.

[33] Jollie M (1977a) A contribution to the morphology and phylogeny of the Falconiformes. Evolutionary Theory 2: 209–300.

[34] Fisher HI (1946) Adaptations and comparative anatomy of the locomotor apparatus of New World vultures. American Midland Naturalist 35: 545–727.

[35] Jollie M (1977b) A contribution to the morphology and phylogeny of the Falconiformes. Evolutionary Theory 3: 1–141.

[36] DeBoer LEM, Sinoo RP (1984) A karyological study of Accipitridae (Aves: Falconiformes) with karyotypic descriptions of 16 species new to cytology. Genetica 65: 89–107.

[37] Wink M (1995) Phylogeny of Old and New World vultures (Aves: Accipitridae and Cathartidae) inferred from nucleotide sequences of the mitochondria1 cytochrome b gene. Z. Naturforsch. 50c: 868–882. 10.1515/znc-1995-11-1220 8561830

[38] Wink M, Sauer-Grth H (2004) Phylogenetic relationships in diurnal raptors based on nucleotide sequences of mitochondrial and nuclear marker genes. In: Chancellor R, Meyburg BU, editors. Raptors worldwide. Berlin: World working group on birds of prey and owls. 483–498.

[39] Brown LH, Amadon D (1968) Eagles, hawks and falcons of the world. London: Country Life Books. 945 p.

